# A Comparative Study of 25 (OH) Vitamin D Serum Levels in Patients with Multiple Sclerosis and Control Group in Isfahan, Iran

**Published:** 2010

**Authors:** Vahid Shaygannejad, Khodayar Golabchi, Sepehr Haghighi, Hamed Dehghan, Amin Moshayedi

**Affiliations:** 1 Department of Neurology, Medical School, Isfahan University of Medical Sciences; Isfahan Neuroscience Research Center, Isfahan University of Medical Sciences, Iran; 2 Student, Isfahan Neuroscience Research Center, Isfahan University of Medical Sciences, Iran

**Keywords:** Serum 25(OH) vitamin D level, Multiple sclerosis, 25(OH) vitamin D

## Abstract

**Objectives:**

There is no study about correlation between vitamin D serum level and multiple sclerosis (MS) in Iran. So in this survey, we investigated the serum level of 25-hydroxy vitamin D in MS patients and compared it with controls in central region of Iran, an area with medium to high risk for MS disease, in spite of high sun exposure.

**Methods:**

A case-control study was conducted from July 1, 2008 to July 31, 2009. We enrolled 50 definitive MS patients, according to McDonald’s criteria as case group and 50 matched controls. Our age limits were 15 to 55 years and those patients with estimated expanded disability status scale less than 5 were introduced to the study. We measured serum level of 25-hydroxy vitamin D and compared them between the two groups.

**Results:**

We gathered 42 females and 8 males as case group and the same numbers as control group without significant age difference. The mean serum level of 25-hydroxy vitamin D in case and control groups were 48 and 62 nmol/L, respectively, and the difference was significant (P=0.036). Also, our study showed significant discrepancy between the two groups according to the rate of deficiency, insufficiency and normal range of vitamin D categories (P=0.021).

**Conclusions:**

We found the same results as those studies carried out in Europe and North America; i.e., lower serum vitamin D level in MS patients than that in normal population, in spite of sufficient sun exposure in Isfahan region.

## INTRODUCTION

Multiple sclerosis (MS) is a chronic probably with autoimmune basis and uncertain etiology characterized by demyelinative and inflammatory physiopathology causing axonal damages.^1^,^2^ This disease affects females more than males with ordinary 10 years relapsing-remitting course and secondary progressive phase.^2^ On histological basis, T helper cells type 1 (Th1) is the most responsible cause for pathologies seen in MS.^1^–^3^ There are multiple risk factors for MS in a complex relationship with each other from genetic ones to environmental factors but exact mechanism leading to MS has remained unclear yet.^4^,^5^ From guessed environmental risk factors for MS, past infection with EBV, smoking and low serum level of vitamin D are more favorable.^6^,^7^ The most important vitamin in calcium and phosphor homeostasis is vitamin D. This vitamin has proposed potential power for modulating immune system.^4^,^8^ This is achieved via enhancing the phagocytic function of monocytes^9^ and decreasing the secretion of IL-6, IL-12, TNF-α and PGE2 by monocytes.^10^ Also, immature or semi mature APC’s cannot mature in presence of 1,25 (OH) _2_D.^8^ Many studies showed a role of paracrine function and nerve growth factor synthesis of vitamin D in CNS and also, showed that deficiency of this vitamin in pregnancy period can cause neural system disorder in animal cases.^8^,^11^–^14^ Then, vitamin D has a near relation with MS and the results of different studies can be summarized as follows: First, the effect of latitude via exposure to sun light and serum level of vitamin D on MS prevalence, that increases with distance from equator, with only one exception in Norway.^2^,^3^,^8^,^15^ Second, Vander et al. showed that sun exposure in childhood period prevents from MS.^16^ Third, it seems low risk of MS in white population is associated with high serum level of vitamin D or high oral intake via supplements or oily fish in some studies.^2^,^17^–^19^ Fourth, it have been shown that vitamin D can be a protective agent against experimental autoimmune encephalitis in mice (like MS in humans).^4^,^8^ Finally, some scientists administered 100-1700 IU/day vitamin D supplements in MS patients and observed the reduction of relapses.^20^–^22^ The candidate for estimating serum level of vitamin D is 25-hydroxy vitamin D, because of its prolonged half-life.^8^,^23^–^25^ It is used to assess an individual’s overall vitamin D nutritional status, because its formation, unlike that of 1, 25-dihydroxy vitamin D, is not tightly regulated and it has a relatively long half-life (20-60 days). There are differences in optimal level of 25-hydroxy vitamin D but nowadays internationally accepted normal range is between 75 nmol/L and 200 nmol/L, and we consider serum level below 75 nmol/L and below 2 nmol/L as insufficiency and deficiency, respectively.^4^,^23^,^25^ Even it seems to achieve the prevention of autoimmune diseases, the serum level of vitamin D must be higher than the level required to reduce osteoporosis (higher than 75 nmol/L).^4^ In attention to high prevalence of MS in Iran and especially in Isfahan which is about 43.5/100,000^26^,^27^ and given that Iran is located in an latitude of 32° 50’ and longitude of 51° 43’ with enough sun exposure and because nobody investigated the relationship between vitamin D, as an environmental agent, and MS disease in Iran before, we decided to compare the serum level of 25-hydroxy vitamin D in MS patients and healthy control group in Isfahan region.

## METHODS

Our study was a case-control type. We enrolled randomly 50 patients from definitive MS patients on the basis of their MRI findings and clinical presentations that were recorded in MS society of Isfahan as case group. Also, we included 50 healthy populations randomly from Alzahra hospital’s staff that were matched on sex, age and living style situation to case group as control one. All of them were residents of Isfahan with the age of 15 to 55 years. We included them if they had the following criteria: no known diseases related to vitamin D deficiency such as rickets or parathyroid diseases and no intake of drug or supplement containing vitamin D or calcium. In the next stage, two neurologists estimated expanded disability status scale (EDSS) in MS patients and those with EDSS less than 5 (that had capability to do outdoor activity) were included in our study. We informed all patients about details and goals of this study before the start. In attention to exclusion criteria (not to give blood sample or usage of vitamin D supplement during the study period) none of the case and control populations were not excluded from our research. To be sure that sun exposure didn’t influence our analysis, we decided to take blood sample in winter (from December to February). Two ml blood sample was taken from all participants and then, 25-hydroxy vitamin D was measured using proper kit (Immunodiagnostic systems – *ids* – made by UK). All of these stages took place in Khaje Nasir biochemistry and clinical diagnosis laboratory. After gathering information, we divided all subjects into three categories on the base of their 25-hydroxy vitamin D serum level as follows: vitamin D deficient patients (serum level below 25 nmol/L), vitamin D insufficient cases (serum level between 25 and 75 nmol/L) and subjects with normal or higher than normal vitamin D level (serum level higher than 75 nmol/L). The mean 25-hydroxy vitamin D levels in case and control groups were compared with independent t test. The relationship of vitamin D level with other factors was compared by chi-square test.

## RESULTS

**Figure 1:**
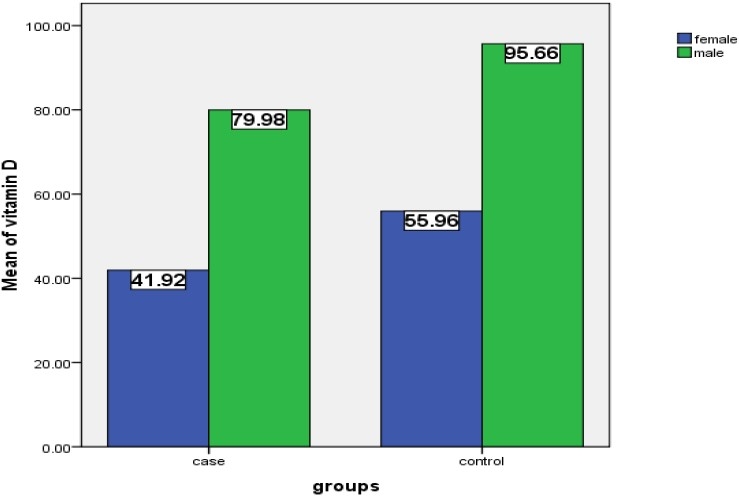
Mean serum levels of vitamin D based on sex in the two groups.

**Figure 2:**
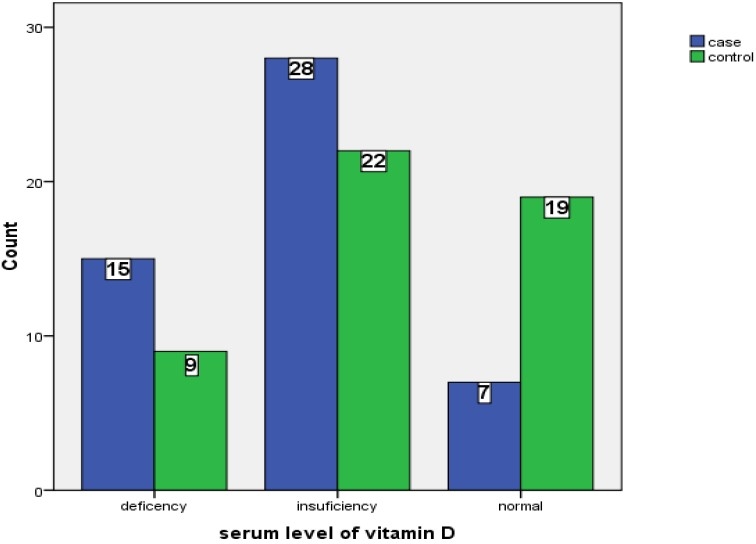
Serum levels of vitamin D in three categories in case and control groups.

This case-control study evaluated 50 MS patients as case group and 50 healthy people as control. The mean age in case and control group was 36.62 and 34.96 years, respectively. Forty two females and 8 males were included in each group. Sex and age was matched between the two groups. The mean levels of vitamin D in the two groups are shown in Figure 1. The mean level of 25-hydroxy vitamin D in case and control groups were 48 and 62 nmol/L, respec-tively, and it showed a significant difference between them (P=0.036).There was not any significant relationship between age and serum level of vitamin D in both groups (P=0.83). The number of patients with vitamin D deficiency, insufficiency and normal range in the case group were 15 (30%), 28 (56%) and 7 (14%), respectively. These numbers for control group were 9 (18%), 22 (44%), 19 (38%), respectively. This study showed a significant relationship between serum levels of vitamin D between the two groups (P=0.021). Serum levels of vitamin D in three categories in the two groups are shown in Figure 2. The mean levels of vitamin D in the case group for deficient, insufficient and normal categories were 13.78, 49.14, and 116.81, respectively and these numbers for control group were 17.44, 53.34, and 93.97, respectively.

## DISCUSSION

We found the same results as studies in Europe and North America, which implicates lower serum vitamin D level in MS patients than in normal population, in spite of sufficient sun exposure in Isfahan region.^2^,^3^,^8^,^15^ The hypothesis that vitamin D deficiency is a risk factor for MS was first proposed over 30 years ago^4^ and gained credibility after the discovery of the immunomodulatory effects of vitamin D.^5^ Moreover, in the past few years, the epidemiological evidence of an increased MS risk among individuals with low vitamin D concentrations has achieved substantial strength. The primary form of vitamin D, colecalciferol (vitamin D3), is available from two sources: skin exposure to ultraviolet B radiation (UVB) in sunlight and diet. UVB in the 290-315 nm range photolysis 7-dehydrocholesterol in the skin to form previtamin D3, which then isomerizes to colecalciferol.^6^ Colecalciferol (and ergocalciferol [vitamin D2]) is also available from fortified foods (e.g., milk, cereal, and some orange juice and cheeses), dark fish (e.g., salmon and tuna), and vitamin supplements (colecalciferol). Relative to sun exposure, diet is a poor source of colecalciferol, providing only 40-400 IU per food serving,^12^ whereas whole-body UVB exposure for 20 min for a light-skinned person during the summer months will produce at least 10,000 IU.^7^,^13^ However, increased skin pigmentation, age, use of sunscreen, built environment, and environmental factors that reduce the strength of UVB reaching the earth’s surface (e.g., winter season, high latitude, pollution, cloud cover, and ozone levels) all contribute to reduce skin colecalciferol production to the point at which diet might become the primary source.^7^–^14^ Because melanin pigment in human skin absorbs UVB,^28^ black people have lower 25 hydroxyvitamin D concentrations than white people, and are often vitamin D deficient.^21^ However, the risk of MS in black people is lower than in white people (because of genetic differences).^28^ Analyses of the relation between vitamin D status and MS risk are therefore better done separately within each race/ethnic group. The importance of age of exposure and seasonality is uncertain. Studies in migrants implicate postnatal environmental exposures, but do not exclude prenatal effects.^15^,^29^ Blood concentrations of 25-hydroxyvitamin D fluctuate with season, but the etiological relevance of these fluctuations to MS risk is unknown. The reasons why vitamin D deficiency is thought to be a risk factor for MS are as follows: First, MS frequency increases with increasing latitude, which is strongly inversely correlated with duration and intensity of UVB from sunlight and vitamin D concentrations.^28^,^30^–^35^ Second, prevalence of MS is lower than expected at high latitudes in populations with high consumption of vitamin-D-rich fatty fish^4^,^35^,^36^. Third, MS risk seems to decrease with migration from high to low latitudes.^15^ If vitamin D had any effect on MS risk, we would expect MS incidence to decrease with increasing 25-hydroxyvitamin D concentrations. The only study satisfying this condition used a nested case-control design to sample an underlying prospective cohort comprising over 7 million individuals who served in the US military and had at least two serum samples stored in the US Department of Defense Serum Repository.^18^ The study concluded that serum concentration of 25 hydroxyvitamin D in healthy young white adults is an important predictor of their risk of developing MS, independently from their place of birth and latitude of residence during childhood.^18^ In a prospective investigation comprising approximately 200,000 women in the USA, vitamin D intake was measured every 4 years by a comprehensive semi-quantitative food frequency questionnaire.^37^ The incidence of MS during the 30-year follow-up decreased with increasing vitamin D intake (P=0.03 for trend) and was 33% lower among women in the highest quintile of vitamin D intake versus those in the lowest quintile.^17^ Furthermore, MS incidence was 41% lower among women taking 400 IU per day or more from supplements compared with non-users.^17^ Attempts to relate vitamin D intake to MS risk have also been made in casecontrol studies. In Norway, a study comprising 119 MS cases and 251 controls living above the Arctic Circle, where fatty fish is a major contributor to vitamin D intake, reported a lower risk of MS for individuals who ate fish three or more times per week at 16–20 years of age compared with those with a lower consumption (odds ratio=0.57 [95%CI 0.33-0.93]; P=0.024).^19^ Supplementation with codliver oil was also associated with lower risk of MS, but only among individuals with low summer outdoor activity. In relation to sun exposure and related measures, one of these studies, which comprised 136 individuals with MS and 272 age and sex-matched controls in Tasmania,^16^ collected information on sun exposure at different ages. The odds ratios of MS among individuals who reported spending at least 2 hours per day in the sun during holidays and at weekends at ages 6-10 years were 0.47 (95%CI 0.26-0.84) for winter and 0.50 (0.24-1.02) for exposure; inverse but weaker associations were observed with sun exposure at older ages. Non-melanoma skin cancer and, less strongly, melanoma are more common in individuals with high levels of sun exposure. Thus, if vitamin D was protective, these cancers would be expected to be rare among individuals with MS. In fact, a lower than expected occurrence of skin cancer was reported among individuals with MS in a study in the UK.^38^ Month of birth has also been suggested as a factor that affects MS risk. In a pooled analysis of data from Canada, UK, Denmark, and Sweden including more than 40,000 individuals with MS, significantly fewer (8.5%) people with MS were born in November and significantly more (9.1%) were born in May.^39^ This finding suggests that prenatal exposures or exposures in the first months of life could be important in MS etiology, but the link to vitamin D is unclear. Genes also can affect vitamin D metabolism, skin color, and behavior, all of which can influence circulating 25-hydroxyvitamin D concentrations. Furthermore, genetic variations in vitamin-D-related and other genes might influence the effects of vitamin D on the immune system. Therefore, genetic variations in vitamin-D-related genes might also affect MS risk, either directly or by modifying the effects of vitamin D.^40^

Our implications for future research and MS prevention and according to the basis of the results of the only longitudinal study of serum 25-hydroxyvitamin D and MS onset,^18^ and assum-ing that these results are unbiased and vitamin D is truly protective against MS, over 70% of MS cases in the USA and Europe could be prevented by increasing the serum 25-hydroxyvitamin D concentration of adolescents and young adults to above 100 nmol/L.^41^,^42^ These concentrations are commonly found only in individuals with outdoor lifestyles in sunny regions, but could be reached in most people by taking 1000-4000 IU colecalciferol daily.^8^,^25^,^43^,^44^ Although these doses are largely considered safe and potentially beneficial for other outcomes,^8^,^19^ confirmation of safety and efficacy in a large randomized trial is needed before making general recommendations. One option would be a national or multinational study based on randomization of school districts or other suitable units in regions of low sunlight intensity, perhaps including multiple outcomes that might be affected by vitamin D, such as diabetes, obesity, respiratory infections, and asthma.^45^,^46^ Alternatively, high-risk first-degree relatives of individu-als with MS could be targeted in a smaller trial,^47^ although randomization and compliance could be more challenging. In either design, contamination of the control group would be a potential concern.

## CONCLUSIONS

In conclusion, this study showed that serum vitamin D deficiency might be a potential environmental predisposing factor for developing multiple sclerosis in our region, and need to follow factors that cause this problem including dietary, genetic, and other ones. Whereas future observational epidemiological studies and genetic and molecular investigations will be useful to strengthen and refine the hypothesis, it might be necessary to do a large randomized trial to establish the safety and efficacy needed to promote large-scale vitamin D supplementation. A test of the hypothesis that vitamin D could reduce MS risk will require the administration of relatively high doses of vitamin D to hundreds of thousands of young adults for several years, and careful monitoring for unforeseen adverse effects is mandatory. We suggest that an international multidisciplinary working group to be set up to oversee the design of future prevention or supplementation studies. Furthermore, screening of serum 25-hydroxyvitamin D concentrations is likely to identify a large proportion of patients who are vitamin D deficient or insufficient, and who might benefit from vitamin D supplementation for prevention of osteoporosis and other complications, especially multiple sclerosis.
